# The Princess Christian Hospital Train

**Published:** 1900-05-26

**Authors:** William Stokes

**Affiliations:** Surgeon in Ordinary to H.M. the Queen in Ireland, Consulting Surgeon to the Field Forces, S. Africa.


### The Institutional Workshop.

### THE PRINCESS CHRISTIAN HOSPITAL TRAIN.

### INITIAL JOURNEY TO LADYSMITH.

By Sir WILLIAM STOKES, F.R.C.S.I, Surgeon in Ordinary to H.M. the Queen in Ireland, Consulting Surgeon to the Field Forces, S. Africa.

AMONG the many philanthropic efforts that have been made in England, in the Colonies, and America to promote the comfort and welfare of our brave sick and wounded soldiers serving in the present war, there is none that will be remembered with greater gratitude than the establishment by the Central British Red Cross Committee of the hospital train, which, in consequence of the national support given, and keen interest shown in it by H.R.H. the Princess Christian, has been appropriately named after her. It is interesting to note that the first train that went from Durban to Ladysmith after the siege was that named after the Princess Christian, and I deem it a fortunate circumstance for myself that, thanks to Sir John Furley's kindness, I was a passenger in it on that interesting occasion, and can, therefore, bear testimony to the admirable way the train was equipped, to the intelligent thoughtfulness that was manifested in every department to secure the comfort and care of the sick and wounded, and the skill that was displayed in the practical arrangements made to provide for emergencies. A few facts connected with the organisation and management of the Princess Christian hospital train may be mentioned.

The greater part of the cost of the train has been defrayed by the town of Windsor. The contract was taken by the Military Equipment Company, London, and it was built by the Birmingham Railway Wagon Works Company, and completely built, fitted, and shipped in the short space of twelve weeks. To Sir John Furley is due the credit of designing the train and superintending all the details connected with it, having associated with him Mr. Fieldhouse, a director of the Military Equipment Company. The hospital train consists of seven carriages, each 36 ft. in length, with a continuous passage through the centre. The first carriage is divided into three compartments, the first being the linen closet, the second a room with two beds for nurses, and the third is set aside for two invalid officers. The second carriage is also divided into three compartments, one for two medical officers, another a dining-room for the staff, and, lastly, a dispensary. The third, fourth, fifth, and sixth carriages are for 72 non-commissioned officers and men. There are also 15 orderlies and a cook. The seventh carriage contains the guard's van, with a good kitchen on one side and a pantry on the other. There are seven closets and lavatories, and seven stores and an abundant supply of water throughout the train. The ventilation throughout is admirable, and portable steps are provided, and awnings to shelter the patients on their arrival and departure. The Union Jack and the Red Cross flags decorate the head of the train, and on both sides of each carriage a red cross on a white ground is painted. The beds, which are of iron with a wire netting supporting a hair mattress, are so arranged that they can easily be taken down, and the patient placed on it and thus lifted into the carriage. They are then raised and fixed on the supporting brackets. As regards the comforts, conveniences, utilisation of every available space, and the cheerful, bright appearance of all the surroundings, fittings and furniture of the carriages, one is forcibly reminded of the similar arrangements which are found in the cabins of a first-class yacht. Each patient on leaving the train at Durban and going on board an hospital ship, or one bound for England, is presented with a bag containing pyjamas, socks, handkerchiefs, a brush and comb, sponge, &c.—a generous gift which is largely appreciated.

On March 19th, at 3.45 a.m., accompanied by Lieut.-General Sir Francis Clery, K.C.B., and Major Wynn, A.D.C., I left the Mooi River Station, and was comfortably accommodated in one of the carriages, and after about two hours' good sleep, and passing Chieveley and Frere, reached Colenso, and got for the first time a view of the famous Tugela River, the scene of so much tragedy, disaster, and reverse. Here we halted, and received the unpleasant information that we could not proceed for at least an hour as the trestle bridge across the river was not yet completed. This has been erected close to the old bridge that had been blown up by the Boers; and certainly this unhappy and destructive operation was carried out very thoroughly. It was a sad sight to see such a noble structure so utterly destroyed.

It was not very reassuring to learn that the new bridge was not yet completed, and we failed also to learn if steps had been taken to ascertain if its strength was sufficient to enable our train to cross over. After a comfortable breakfast in the dining-room of the train we proceeded slowly and cautiously across the bridge, and I confess to having felt some apprehension while crossing so apparently frail a structure, which feeling was not diminished when the train came suddenly to a standstill in the centre of the bridge. While waiting there it was not a little disturbing to our feelings to hear the woodwork creaking and groaning under the weight of our train, with an obligato accompaniment produced by the noise of the rushing waters beneath us. Not knowing the cause of the delay, we had here a veritable *mauvais quart d'heure*. However, on inquiring subsequently the cause of the stoppage we were informed that it was in consequence of Major Brazier Creagh, R.A.M.C. (among whose many and varied accomplishments the art of photography must be included) being desirous of taking a view of the first train crossing the new bridge, which it was suggested should be forwarded to H R.H. the Princess Christian. This, I hope, has been done. Ultimately we got safely across and thankfully breathed freely again.

On the journey to Ladysmith, we passed through, or saw from a short distance, many of the places associated with the war we have been reading and hearing of so long—Colenso, the Tugela, Fort Napier, Grobler's Kloof, Greenhill, Fort Wylie, Pieters Hill, and the Vaal Krantz range of hills—and realised the enormous topographical difficulties that had to be faced in order to effect the relief of Ladysmith; difficulties which few people who have not seen the country can possibly realise. The traces of recent fighting along the route were very sad—houses in ruins, broken wagons, camp débris, skeletons of horses and mules, and, on the crests of the hills, rows of the low, roughly built "sangers" behind and from which the Boers had sent showers of the deadly Mauser bullets, bringing death and disablement to many a gallant soldier in our ranks. Here and there in these "sangers" were gaps or openings, "epaulements" behind which the larger guns were placed, and from which came destructive shells, pom-poms, and shrapnel. Many of the hills were thickly covered with the low, prickly mimosa shrub, which furnished such excellent cover for the Boer marksmen. We also passed the famous unfinished dam across the Klip River by means of which the Boers hoped to inundate the country round Ladysmith and drown all the inhabitants! There was rather a miscalculation on the Boer engineers in this proposed humane operation, for, as Sir Redvers Buller said to me subsequently, to carry out the object effectively, the height of the dam should have been at least 80 ft.

Shortly after passing this ineffective structure we reached Ladysmith, in which were, of course, many sad evidences of its protracted siege. The people looked half-starved, the women and children especially emaciated and anæmic; houses were broken down, windows smashed, and the tower of the town hall, which had been used as a hospital, in ruins. But still there was less destruction of property than I had anticipated. This was chiefly caused by the Boer fire having been mainly directed across the northern side of the town from Isimbulwana Hill to "Cæsar's Camp," where a large proportion of our forces were located.

I visited Sir Redvers Buller, who with his staff were stationed at the Convent, from which a fine panoramic view could be had of all the surrounding hills previously occupied by the Boers. He was most kind and hospitable, and I had some most interesting conversation with him about the war. Among other things he told me the Boers had recently received large reinforcements at Biggarsberg, about 16 or 18 miles from Ladysmith, and also that he feared there would be trouble from the Boers in Zulu.

I spent the night in the Royal Hotel, which was reopened the day I arrived. As might be expected, things were somewhat uncomfortable—no milk, butter, eggs, coffee, or vegetables to be had for "love or money"! However, my host evidently did his best for my comfort, and, after a fatiguing day, I was glad to get rest in a well-appointed room, well protected against mosquitoes, which are of a particularly blood-thirsty type in this district, and have all the obstinacy, pertinacity, and doggedness that are so conspicuous in the Boer character! I regret I was unable to visit on this occasion any of the positions held by the Boer and our forces respectively in the neighbourhood of Ladysmith, but which I hope to see on a future occasion, as, late at night, I received a telegram from Colonel Galway, C.B., the Principal Medical Officer of Natal, which necessitated an immediate return to Maritzburg to see Count Mataxa, who was reported to be dangerously wounded. Accordingly, I left Ladysmith, bringing with me reminiscences, as interesting as they were agreeable, not only of the district through which I had passed and the places I visited, but also of the initial journey to Ladysmith of the Princess Christian Hospital Train.

It is interesting and gratifying to note that the good and important work this hospital train has been the means of accomplishing may be estimated by the fact that since its initial journey to the present date (April 24th) 70 invalid officers, 7 sick nursing sisters, and 875 non-commissioned officers and men have, without a single fatality, been conveyed from the various hospitals along the lines of communication to Durban. The signal success that has attended this important service is doubtless due to the professional skill and great administrative ability of the medical officers in charge—Surgeon Lieut.-Colonel Forrester (Royal Horse Guards), assisted by Civil Surgeon P. R. Lowe, M.B.Cantab, whose efforts have been as assiduous as they were unremitting.

